# Supplementary material for: Association of Antibiotic Use during the First 6 Months of Life with Body Mass of Children
Source: Antibiotics (Basel). 2022 Apr 11;11(4):507. doi: 10.3390/antibiotics11040507 (PMC9033100; doi:10.3390/antibiotics11040507)
Supplement: Supplementary file 1 [file antibiotics-11-00507-s001.zip › antibiotics-1632021-supplementary.pdf]

Supplementary methods – the additional cohort

The additional cohort included children (1) whose birth weight information was available through the NHSPIC, (2) who completed the first round of the NHSPIC, and (3) who completed at least 1 round from the fourth to the seventh round of the NHSPIC. Children were excluded in they (1) were born before 37 weeks of gestational age, (2) had birth weight less than 2.5 kg or more than 4 kg, or (3) had been admitted to the intensive care unit before 3 months of age. Of the 369,733 children, 97,653 children who had used antibiotics before 3 months of age were assigned to the antibiotic users group, and 272,080 children who had not used antibiotics before 3 months of age were assigned to the antibiotic non-users group as a control group. After 1:1 propensity score matching, 59,443 children were assigned to the antibiotic users group and the same number to the antibiotic non-users group. (Figure S1)

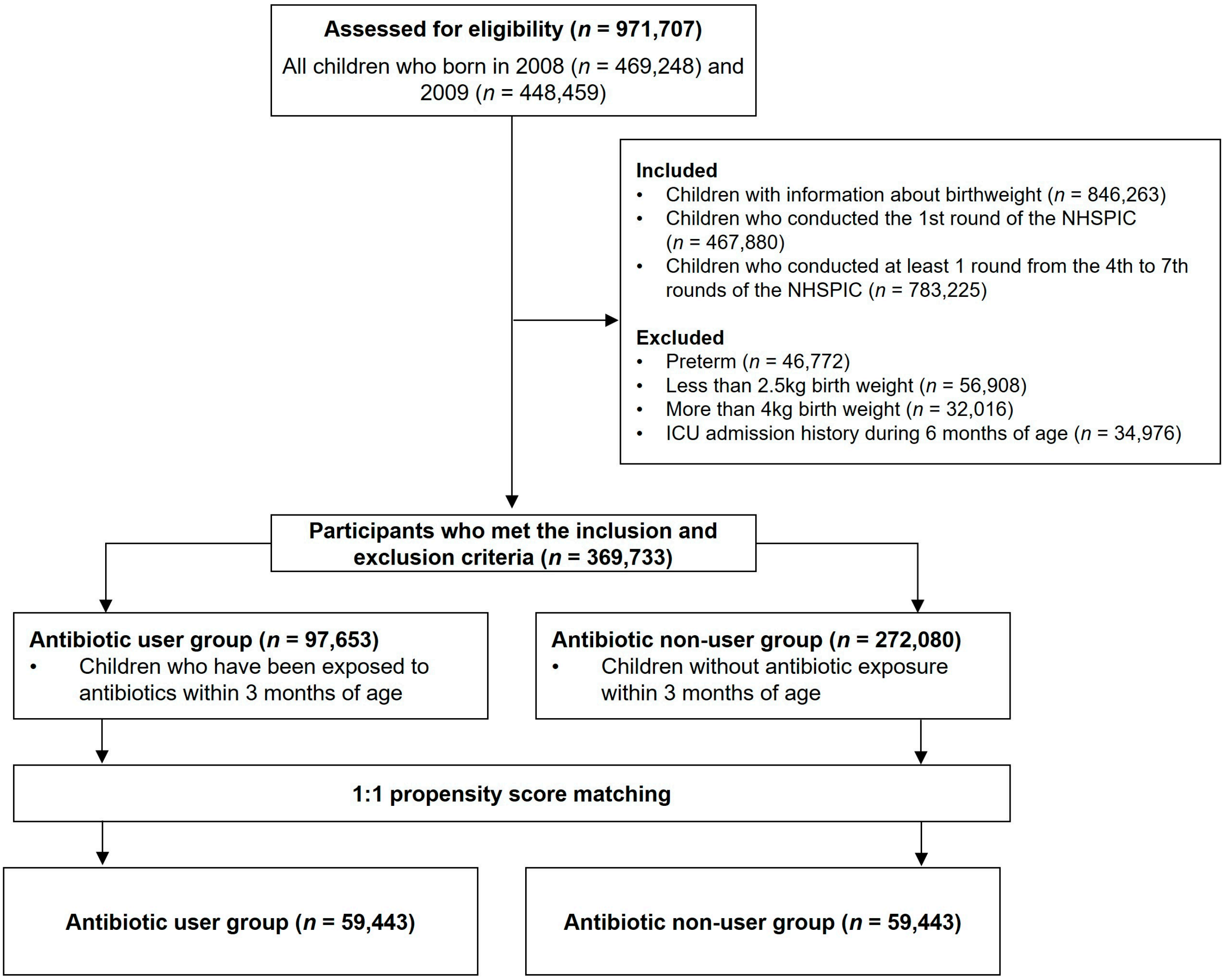

Figure S1. Flow diagram of the additional cohort.

Table S1. Checklist of Recommendations for Reporting of Observational Studies Using the Reporting of Studies Conducted Using Observational Routinely Collected Health Data (RECORD) Guidelines.

	Item No	Recommendation	Reported

Title and abstract	1	(a) Indicate the study's design with a commonly used term in the title or the abstract	Abstract
		(b) Provide in the abstract an informative and balanced summary of what was done and what was found	Abstract
**Introduction**			
Background /rationale	2	Explain the scientific background and rationale for the investigation being reported	Introduction
Objectives	3	State specific objectives, including any prespecified hypotheses	Introduction
**Methods**			
Study design	4	Present key elements of study design early in the paper	Methods - Study Design and Setting
Setting	5	Describe the setting, locations, and relevant dates, including periods of recruitment, exposure, follow-up, and data collection	Methods - Study Design and Setting, Data sources
Participants	6	(a) Give the eligibility criteria, and the sources and methods of selection of participants. Describe methods of follow-up	Methods – Study population and Figure 1
		(b) For matched studies, give matching criteria and number of exposed and unexposed	Methods – Covariates, Statistical analysis; Results - Baseline Characteristics; Tables 1 Table 2, and eTable 2, eTable 3, eTable 4, and eTable 5
Variables	7	Clearly define all outcomes, exposures, predictors, potential confounders, and effect modifiers. Give diagnostic criteria, if applicable	Methods - Data Sources; Exposure; Outcome; Covariates
Data sources /measurement	8	For each variable of interest, give sources of data and details of methods of assessment (measurement). Describe comparability of assessment methods if there is more than one group	Methods - Data Sources;
Bias	9	Describe any efforts to address potential sources of bias	Methods – Covariates; Statistical analysis;<br>subgroup analysis and post-hoc analysis<br>Discussion
Study size	10	Explain how the study size was arrived at	Methods – study population, Figure 1
Quantitative variables	11	Explain how quantitative variables were handled in the analyses. If applicable, describe which groupings were chosen and why	Methods – statistical analysis;<br>subgroup analysis and post-hoc analysis
Statistical methods	12	(a) Describe all statistical methods, including those used to control for confounding	Methods – Covariates; statistical analysis;<br>subgroup analysis and post-hoc analysis
		(b) Describe any methods used to examine subgroups and interactions	Methods – statistical analysis;<br>subgroup analysis and post-hoc analysis
		(c) Explain how missing data were addressed	Table 1 and eTable 4
		(d) If applicable, explain how loss to follow-up was addressed	Not applicable
		(e) Describe any sensitivity analyses	Methods – Study population, subgroup analysis and post-hoc analysis

Results			
Partici-pants	13	(a) Report numbers of individuals at each stage of study, e.g., numbers potentially eligible, examined for eligibility, confirmed eligible, included in the study, completing follow-up, and analyzed	Results - Participants; Figure 1; eFigure 1
		(b) Give reasons for non-participation at each stage	Figure 1
		(c) Consider use of a flow diagram	Figure 1
Descrip-tive data	14	(a) Give characteristics of study participants (e.g. demographic, clinical, social) and information on exposures and potential confounders	Results - Participants; Table 1, Table 2, eTable 3, eTable 4, and eTable 5
		(b) Indicate number of participants with missing data for each variable of interest	Methods – Table 1, eTable 4
		(c) Summarize follow-up time (e.g. average and total amount)	Methods – Data sources
Outcome data	15	Report numbers of outcome events or summary measures over time	Results – Main outcome, additional outcome, Table 3, Table 5, eTable 6 and eTable 7
Main results	16	(a) Give unadjusted estimates and, if applicable, confounder-adjusted estimates and their precision (e.g., 95% confidence interval). Make clear which confounders were adjusted for and why they were included	Results - Main outcome, additional outcome, Table 3, Table 5, eTable 6 and eTable 7
		(b) Report category boundaries when continuous variables were categorized	Results – Table 3, Table 4, Table 5, Table 6, eTable 6 and eTable 7
		(c) If relevant, consider translating estimates of relative risk into absolute risk for a meaningful time period	Not applicable
Other analyses	17	Report other analyses done, e.g., analyses of subgroups and interactions, and sensitivity analyses	Results – Figure 4, eTable 8, eTable 9, and eTable 10
**Discussion**			
Key result	18	Summarize key results with reference to study objectives	Discussion
Limitation	19	Discuss limitations of the study, taking into account sources of potential bias or imprecision. Discuss both direction and magnitude of any potential bias	Discussion
Interpretation	20	Give a cautious overall interpretation of results considering objectives, limitations, multiplicity of analyses, results from similar studies, and other relevant evidence	Discussion
Generalizability	21	Discuss the generalizability (external validity) of the study results	Discussion
Other information			
Funding	22	Give the source of funding and the role of the funders for the present study and, if applicable, for the original study on which the present article is based	Article Information

Table S2. Covariables in the propensity score matching of the main cohort.

Characteristic	Database	Variables
**Demographics**	NHIS	Age, sex, income quintile, residence at birth

	NHSPIC	Birth weight, type of feeding (breastmilk, formula, mixed, or special milk) until 3-6 months of life, whether infant received additional complementary feeding before 3 months of age
**Comorbidities**		
Health Care Use	NHIS	Any hospitalization, ICU admission
Perinatal condition	NHIS (ICD-10 code)	
	P00–P04	Fetus and newborn affected by maternal factors and by complications of pregnancy, labor and delivery
	P05–P08	Disorders related to length of gestation and fetal growth
	P10–P15	Birth trauma
	P20–P29	Respiratory and cardiovascular disorders specific to the perinatal period
	P35–P39	Infections specific to the perinatal period
	P50–P61	Hemorrhagic and hematological disorders of fetus and newborn
	P70–P74	Transitory endocrine and metabolic disorders specific to fetus and newborn
	P75–P78	Digestive system disorders of fetus and newborn
	P80–P83	Conditions involving the integument and temperature regulation of fetus and newborn
	P90–P96	Other disorders originating in the perinatal period
	Q00–Q99	Congenital malformations, deformations and chromosomal abnormalities
Prevalent disease exposure	NHIS (ICD-10 code)	
	Z38.0	Singleton, born in hospital
	A08.4	Viral intestinal infection, unspecified
	A09.0	Other and unspecified gastroenteritis and colitis of infectious origin
	A09.9	Gastroenteritis and colitis of unspecified origin
	B37.0	Candidal stomatitis
	H10.0	Mucopurulent conjunctivitis
	H10.2	Other acute conjunctivitis
	H10.3	Acute conjunctivitis
	H10.9	Conjunctivitis, unspecified
	H66.0	Acute suppurative otitis media
	J00	Acute nasopharyngitis
	J10.0	Influenza due to other identified influenza virus with pneumonia
	J01.9	Acute sinusitis, unspecified
	J02.9	Acute pharyngitis, unspecified
	J03.9	Acute tonsillitis, unspecified
	J06.0	Acute laryngopharyngitis
	J06.8	Other acute upper respiratory infections of multiple sites
	J06.9	Acute upper respiratory infection, unspecified
	J18.9	Pneumonia, unspecified
	J20.9	Acute bronchitis
	J21.9	Acute bronchiolitis
	J22.0	Unspecified acute lower respiratory infection
	J30.4	Allergic rhinitis
	J45.9	Other and unspecified asthma
	K21.9	Gastro-esophageal reflux disease without esophagitis
	K30	Functional dyspepsia
	K52.9	Noninfective gastroenteritis and colitis, unspecified
	K59.0	Constipation
	K59.1	Functional diarrhea
	K59.9	Functional intestinal disorder, unspecified

	L01.0	Impetigo [any organism] [any site]
	L20.8	Other atopic dermatitis
	L20.9	Atopic dermatitis
	L21.1	Seborrheic infantile dermatitis
	L21.9	Seborrheic dermatitis
	L22	Diaper dermatitis
	L23.9	Allergic contact dermatitis
	L24.9	Irritant contact dermatitis, unspecified cause
	L30.9	Dermatitis
	L98.0	Pyogenic granuloma
	N39.0	Urinary tract infection, site not specified
	R10.4	Other and unspecified abdominal pain
	R11	Nausea and vomiting
	R50.9	Fever, unspecified
	R68.1	Nonspecific symptoms peculiar to infancy
	E40-E46	Malnutrition
Drug exposure	NHIS (drug classification code)	
	112	Sedative-hypnotics
	113	Antiepileptics
	114	Antipyretics
	117	Psycho-nervous system drug
	123	Automatic nervous system drugs
	124	Antispasmodics
	141	Antihistamine
	149	Other allergic drug
	211–219	Circulatory system drugs
	221–229	Respiratory system drugs
	231–239	Digestive system drugs
	241–249	Hormone drugs
	245	Steroid

Table S3. Basic clinical characteristics of children in the main cohort[a].

Clinical Characteristics	Observed Data (n = 369,578)				Matched Data (n = 145,966)[b]		
	Antibiotic, n (%)[c]		Standardized Difference %[d]		Antibiotic, n (%)[c]		Standardized Difference %[d]
	Users (n = 203,073)	Non-users (n = 166,505)			Users (n = 72,983)	Non-users (n = 72,983)	
Hospital utilization							
No. hospitalized	50,256 (24.7)	12,045 (7.2)	48.6		9402 (12.9)	8790 (12.0)	1.1
No. visiting ER	16,360 (8.1)	6399 (3.8)	17.4		3906 (5.4)	3883 (5.3)	0.0
Certain conditions (ICD-10 code) originating in the perinatal period							
Fetus and newborn affected by maternal factors and by complications of pregnancy, labor and delivery	4799 (2.4)	2396 (1.4)	6.8		1500 (2.1)	1492 (2.0)	0.1
Disorders related to length of gestation and fetal growth	875 (0.4)	494 (0.3)	2.3		272 (0.4)	245 (0.3)	0.6
Birth trauma	1912 (0.9)	1316 (0.8)	1.6		664 (0.9)	664 (0.9)	0.0

Respiratory and cardiovascular disorders specific to the perinatal period	10,677 (5.3)	4936 (3.0)	11.8		3180 (4.4)	3108 (4.3)	0.5
Respiratory and cardiovascular disorders specific to the perinatal period	33,680 (16.6)	18,593 (11.2)	15.8		10,209 (14.0)	10,164 (13.9)	0.2
Hemorrhagic and hematological disorders of fetus and newborn	67,110 (33.0)	50,367 (30.2)	6.1		23,356 (32.0)	23,292 (31.9)	0.2
Transitory endocrine and metabolic disorders specific to fetus and newborn	6028 (3.0)	3580 (2.2)	5.4		1947 (2.7)	1876 (2.6)	0.6
Digestive system disorders of fetus and newborn	6473 (3.2)	4151 (2.5)	4.2		2153 (3.0)	2182 (3.0)	0.2
Conditions involving the integument and temperature regulation of fetus and newborn	8174 (4.0)	5944 (3.6)	3.4		2716 (3.7)	2689 (3.7)	0.2
Other disorders originating in the perinatal period	12,174 (6.0)	9496 (5.7)	1.3		4438 (6.1)	4405 (6.0)	0.2
Congenital malformations, deformations and chromosomal abnormalities	21,216 (10.4)	14,131 (8.5)	6.8		6956 (9.5)	7013 (9.6)	0.3
Prevalent diseases (ICD-10 codes) diagnosed before 6 months of age							
Singleton, born in hospital	202,893 (99.9)	163,244 (98.0)	18.6		72,815 (99.8)	72,805 (99.8)	0.1
Viral intestinal infection, unspecified	8904 (4.4)	5202 (3.1)	6.6		2957 (4.1)	3003 (4.1)	0.3
Other and unspecified gastroenteritis and colitis of infectious origin	17,544 (8.6)	8203 (4.9)	14.8		5134 (7.0)	5122 (7.0)	0.1
Gastroenteritis and colitis of unspecified origin	18,590 (9.2)	9019 (5.4)	14.5		5574 (7.6)	5584 (7.7)	0.1
Candidal stomatitis	5026 (2.5)	2481 (1.5)	7.3		1320 (1.8)	1373 (1.9)	0.5
Mucopurulent conjunctivitis	4950 (2.4)	1790 (1.1)	10.5		1235 (1.7)	1218 (1.7)	0.2
Other acute conjunctivitis	6154 (3.0)	2657 (1.6)	9.4		1695 (2.3)	1691 (2.3)	0.0
Acute conjunctivitis	11,520 (5.7)	5234 (3.1)	12.2		3351 (4.6)	3375 (4.6)	0.2
Conjunctivitis, unspecified	11,828 (5.8)	5842 (3.5)	11.0		3566 (4.9)	3620 (5.0)	0.4
Acute suppurative otitis media	21,733 (10.7)	430 (0.3)	47.1		468 (0.6)	412 (0.6)	0.3
Acute nasopharyngitis	80,109 (39.4)	46,375 (27.9)	24.7		25,304 (34.7)	22,595 (31.0)	0.9

Influenza due to other identified influenza virus with pneumonia	4927 (2.4)	590 (0.4)	17.8		536 (0.7)	506 (0.7)	0.4
Acute sinusitis, unspecified	16,024 (7.9)	2419 (1.5)	30.8		2174 (3.0)	2130 (2.9)	0.3
Acute pharyngitis, unspecified	36,219 (17.8)	15,692 (9.4)	24.6		10,337 (14.2)	10,439 (14.3)	0.4
Acute tonsillitis, unspecified	17,803 (8.8)	14,436 (8.7)	0.5		6504 (8.9)	6545 (9.0)	0.2
Acute laryngopharyngitis	9648 (4.8)	3193 (1.9)	15.8		2322 (3.2)	2335 (3.2)	0.1
Other acute upper respiratory infections of multiple sites	8741 (4.3)	3646 (2.2)	11.9		2347 (3.2)	2375 (3.3)	0.2
Acute upper respiratory infection, unspecified	67,628 (33.3)	35,140 (21.1)	27.7		20,610 (28.2)	21,043 (28.8)	1.3
Pneumonia, unspecified	12,012 (5.9)	399 (0.2)	33.3		424 (0.6)	380 (0.5)	0.4
Acute bronchitis	77,144 (38.0)	14,992 (9.0)	72.7		13,859 (19.0)	13,539 (18.6)	1.1
Acute bronchiolitis	58,490 (28.8)	8549 (5.1)	66.3		8356 (11.4)	7895 (10.8)	1.8
Unspecified acute lower respiratory infection	4818 (2.4)	813 (0.5)	15.8		718 (1.0)	721 (1.0)	0.0
Allergic rhinitis	9333 (4.6)	3218 (1.9)	15.0		2192 (3.0)	2283 (3.1)	0.7
Other and unspecified asthma	11,597 (5.7)	1051 (0.6)	29.3		1020 (1.4)	970 (1.3)	0.4
Gastro-esophageal reflux disease without esophagitis	5853 (2.9)	3882 (2.3)	3.4		2028 (2.8)	2090 (2.9)	0.5
Functional dyspepsia	5213 (2.6)	3963 (2.4)	1.3		1967 (2.7)	2012 (2.8)	0.4
Noninfective gastroenteritis and colitis, unspecified	13,931 (6.9)	8224 (4.9)	8.1		4620 (6.3)	4697 (6.4)	0.4
Constipation	10,322 (5.1)	7752 (4.7)	2.1		3684 (5.0)	3782 (5.2)	0.6
Functional diarrhea	4380 (2.2)	3693 (1.6)	3.9		1473 (2.0)	1498 (2.1)	0.3
Functional intestinal disorder, unspecified	7322 (3.6)	5054 (3.0)	3.2		2558 (3.5)	2620 (3.6)	0.5
Impetigo [any organism] [any site]	4655 (2.3)	2352 (1.4)	6.5		1461 (2.0)	1438 (2.0)	0.2
Other atopic dermatitis	13,350 (6.6)	11,162 (6.7)	0.5		5122 (7.0)	5070 (6.9)	0.3
Atopic dermatitis	37,833 (18.6)	31,269 (18.8)	0.3		14,229 (19.5)	14,242 (19.5)	0.0
Seborrheic infantile dermatitis	5186 (2.6)	4736 (2.8)	1.9		2035 (2.8)	2046 (2.8)	0.1
Seborrheic dermatitis	4684 (2.3)	3,934 (2.4)	0.4		2035 (2.8)	2046 (2.8)	0.2
Diaper dermatitis	17,806 (8.8)	14,436 (8.7)	0.5		6504 (8.9)	6545 (9.0)	0.2
Allergic contact dermatitis	12,138 (6.0)	9821 (5.9)	0.3		4556 (6.2)	4539 (6.2)	0.1
Irritant contact dermatitis, unspecified cause	4916 (2.4)	4183 (2.5)	0.7		1892 (2.6)	1866 (2.6)	0.2

Dermatitis	10,211 (5.0)	8702 (5.2)	0.9		3893 (5.3)	3920 (5.4)	0.2
Urinary tract infection, site not specified	9203 (4.5)	1193 (0.7)	24.1		1075 (1.5)	1045 (1.4)	0.3
Other and unspecified abdominal pain	3318 (1.6)	2183 (1.3)	2.6		1109 (1.5)	1145 (1.6)	0.4
Nausea and vomiting	7569 (3.7)	5058 (3.0)	3.8		2604 (3.6)	2646 (3.6)	0.3
Fever, unspecified	15,068 (7.4)	7366 (4.4)	12.6		4215 (5.8)	4180 (5.7)	0.2
Nonspecific symptoms peculiar to infancy	3081 (1.5)	2720 (1.6)	0.9		1171 (1.6)	1207 (1.7)	0.4
Malnutrition	9 (0.0)	10 (0.0)	0.0		4 (0.0)	6 (0.0)	0.0
Drug (drug classification code) used before 6 months of age							
Sedative- hypnotics	8642 (4.3)	4183 (2.5)	9.6		2537 (3.5)	2532 (3.5)	0.0
Antiepileptics	66 (0.0)	25 (0.0)	1.2		15 (0.0)	16 (0.0)	0.1
Antipyretics	123,846 (61.0)	47,328 (28.4)	69.1		32,519 (44.6)	32,669 (44.8)	0.4
Psycho-nervous system drug	26,818 (13.2)	8887 (5.3)	27.6		6175 (8.5)	6217 (8.5)	0.2
Automatic nervous system drugs	196 (0.1)	67 (0.0)	2.1		40 (0.1)	43 (0.1)	0.2
Antispasmodics	9456 (4.7)	3431 (2.1)	14.6		2340 (3.2)	2401 (3.3)	0.5
Antihistamine	156,558 (77.1)	60,913 (36.6)	89.6		43,719 (59.9)	44,398 (60.8)	2.1
Other allergic drug	3308 (1.6)	528 (0.3)	13.3		454 (0.6)	427 (0.6)	0.4
Circulatory system drugs	24,929 (12.3)	2743 (1.6)	42.6		2571 (3.5)	2423 (3.3)	0.8
Respiratory system drugs	174,725 (86.0)	69,910 (42.0)	103.2		50,853 (69.7)	51,580 (70.7)	2.3
Digestive system drugs	166,479 (82.0)	69,225 (41.6)	91.3		47,331 (64.9)	47,942 (65.7)	1.9
Hormone drugs	25,110 (12.4)	2890 (1.7)	42.3		2641 (3.6)	2493 (3.4)	0.8
Steroid	24,929 (12.3)	2743 (1.6)	42.6		2571 (3.5)	2423 (3.3)	0.8

Abbreviations, N, Number; ICD, International Classification of Diseases; ER, Emergency room. [a] Unless otherwise specified, baseline characteristics were assessed on the day of birth. [b] Propensity score matching (1:1) was performed to reduce bias for the selection of the comparison group. Matching was performed by the Mahalanobis algorithm with a caliper of 0.01 using multivariable logistic regression with 79 previously chosen covariates as defined in Table S2. [c] Values are reported as No. (%) unless otherwise indicated. [d] The difference between the groups divided by the pooled standard deviation; a value greater than 10% is interpreted as a meaningful difference.

Table S4. Basic sociodemographic characteristics of children in the additional cohort[a].

Sociodemographic Characteristics	Observed Data (n = 369,733)				Matched Data (n = 118,886)[b]		
	Antibiotic, n (%)[c]		Standardized Difference % [d]		Antibiotic, n (%)[c]		Standardized Difference % [d]
	Users (n = 97,653)	Non-users (n = 272,080)			Users (n = 59,443)	Non-users (n = 59,443)	
Sex							
Girl	43,644 (44.7)	136,398 (50.1)	10.8		27,686 (46.6)	27,424 (46.1)	0.1
Boy	54,009 (55.3)	135,682 (49.9)			31,757 (53.4)	32,019 (53.9)	
Residence at birth[e]							
Seoul	21,494 (22.0)	69,618 (25.6)	6.3		13,925 (23.4)	14,429 (24.3)	0.9
Metropolitan	24,258 (24.8)	62,609 (23.0)			15,043 (25.3)	13,849 (23.3)	
Urban	39,426 (40.4)	106,523 (39.2)			23,760 (40.0)	24,085 (40.5)	

Rural	11,740 (12.0)	30,779 (11.3)			6715 (11.3)	7080 (11.9)	
Birth year							
2008	49,127 (50.3)	128,986 (47.4)			29,645 (49.9)	29,714 (50.0)	0.2
2009	48,526 (49.7)	143,094 (52.6)			29,798 (50.1)	29,729 (50.0)	
Birth weight, kg (SD)[f]	3.24 (0.32)	3.23 (0.32)	3.6		3.24 (0.32)	3.24 (0.32)	0.2
Type of feeding[g]							
Only breast-feeding	42,810 (43.8)	125,386 (47.4)	1.6		26,737 (45.0)	27,310 (45.9)	1.1
Only formula milk	26,070 (36.9)	91,624 (33.7)			21,367 (35.9)	20,715 (34.8)	
Mixed	17,890 (18.3)	52,963 (19.5)			11,086 (18.6)	11,177 (18.8)	
Special milk	469 (0.5)	942 (0.3)			253 (0.4)	241 (0.4)	
Additional complementary feeding before 3 months of age[h]							
Yes	43,081 (44.1)	114,529 (42.1)	4.1		26,099 (43.9)	26,073 (43.9)	0.1
No	53,879 (55.2)	155,740 (57.2)			33,344 (56.1)	33,370 (56.1)	
Economic status[i]							
1 (Lowest)	7758 (7.9)	20,347 (7.5)	5.6		4742 (8.0)	4814 (8.1)	0.2
2	15,023 (15.4)	38,882 (14.3)			9357 (15.7)	9251 (15.6)	
3 (Middle)	26,762 (27.4)	72,175 (26.5)			16,746 (28.2)	16,821 (28.3)	
4	30,163 (30.9)	87,585 (32.2)			19,339 (32.5)	19,374 (32.6)	
5 (Highest)	14,385 (14.7)	43,878 (16.1)			9259 (15.6)	9183 (15.4)	

Abbreviations, N, Number; SD, standard deviation. [a] Unless otherwise specified, baseline characteristics were assessed on the day of birth. [b] Propensity score matching (1:1) was performed to reduce bias for the selection of the comparison group. Matching was performed by the Mahalanobis algorithm with a caliper of 0.01 using multivariable logistic regression with 79 previously chosen covariates as defined in Table S2. [c] Results are reported as N (%) unless otherwise indicated. [d] Differences greater than 10% were interpreted as meaningful differences. All standardized differences of cohort values were < 0.05. [e] Metropolitan areas were defined as six metropolitan cities (Busan, Incheon, Gwangju, Daegu, Daejeon, and Ulsan), urban areas as cities, and rural areas as non-city areas. Missing data of observed data; user group = 753, non-user group = 2,551 [f] Obtained by the 1st National health screening program of infants and children at 4 to 6 months of age. [g] Obtained by the 1st National health screening program of infants and children at 4 to 6 months of age. Missing data of observed data; user group = 414, non-user group = 1,165, [h] Obtained by the 1st National health screening program of infants and children at 4 to 6 months of age. Missing data of observed data; user group = 693, non-user group = 1,811, [i] Economic status was estimated by the amount of insurance co-payment and classified by quintiles. Missing data of observed data; user group = 3,562, non-user group = 9,213.

Table S5. Basic clinical characteristics of children in the additional cohort[a].

Clinical Characteristics	Observed Data (*n* = 369,733)				Matched Data (*n* = 118,886)[b]		
	Antibiotic, *n* (%)[c]		Standard-ized Difference %[d]		Antibiotic, *n* (%)[c]		Standard-ized Difference %[d]
	Users	Non-users			Users	Non-users	
	(*n* = 97,653)	(*n* = 272,080)			(*n* = 59,443)	(*n* = 59,443)	
Hospital utilization							
No. hospitalized	31,335 (32.1)	19,017 (7.0)	63.6		12,761 (21.5)	11,796 (19.8)	1.4
No. visiting ER	5360 (5.5)	5748 (2.1)	17.6		2377 (4.0)	2398 (4.0)	0.4
Certain conditions (ICD-10 code) originating in the perinatal period, N (%)[c]							
Fetus and newborn affected by maternal factors and by complications of	3466 (3.5)	3732 (1.4)	14.1		1834 (3.1)	1831 (3.1)	0.0

pregnancy, labor and delivery							
Disorders related to length of gestation and fetal growth	536 (0.5)	833 (0.3)	3.8		265 (0.4)	257 (0.4)	0.2
Birth trauma	1050 (1.1)	2182 (0.8)	2.8		621 (1.0)	641 (1.1)	0.35
Respiratory and cardiovascular disorders specific to the perinatal period	7712 (7.9)	7909 (2.9)	22.5		3865 (6.5)	3912 (6.6)	0.4
Respiratory and cardiovascular disorders specific to the perinatal period	21,022 (21.5)	31,268 (11.5)	27.4		11,032 (18.6)	11,335 (19.1)	1.4
Hemorrhagic and hematological disorders of fetus and newborn	34,694 (35.5)	82,835 (30.4)	10.9		20,529 (34.5)	21,316 (35.9)	2.8
Transitory endocrine and metabolic disorders specific to fetus and newborn	3991 (4.1)	5629 (2.1)	11.8		2092 (3.5)	2073 (3.5)	0.2
Digestive system disorders of fetus and newborn	3840 (3.9)	6789 (2.5)	8.1		2110 (3.5)	2159 (3.6)	0.5
Conditions involving the integument and temperature regulation of fetus and newborn	4353 (4.5)	9470 (3.5)	5.0		2505 (4.2)	2497 (4.2)	0.1
Other disorders originating in the perinatal period	6434 (6.6)	15,247 (5.6)	4.1		3837 (6.5)	3953 (6.7)	0.8
Congenital malformations, deformations and chromosomal abnormalities	11,609 (11.9)	23,857 (8.8)	10.3		6555 (11.0)	6699 (11.3)	0.8
Prevalent diseases (ICD-10 codes) diagnosed before 3 months of age							
Singleton, born in hospital	64,656 (66.2)	189,067 (69.5)	6.8		40,048 (67.4)	40,124 (67.5)	0.3
Viral intestinal infection, unspecified	1812 (1.9)	3069 (1.1)	6.0		1014 (1.7)	1030 (1.7)	0.2
Other and unspecified gastroenteritis and colitis of infectious origin	4115 (4.2)	5817 (2.1)	11.8		2116 (3.6)	2179 (3.7)	0.6
Gastroenteritis and colitis of unspecified origin	4612 (4.7)	6634 (2.4)	12.3		2410 (4.1)	2486 (4.2)	0.7
Sepsis, unspecified	2728 (2.8)	278 (0.1)	22.7		332 (0.6)	267 (0.4)	0.9
Candidal stomatitis	1486 (1.5)	2688 (1.0)	4.9		834 (1.4)	840 (1.4)	0.1
Mucopurulent conjunctivitis	2223 (2.3)	2083 (0.8)	12.4		1195 (2.0)	1279 (2.2)	1.2

Other acute conjunctivitis	1934 (2.0)	2661 (1.0)	8.1		1082 (1.8)	1162 (2.0)	1.1
Acute conjunctivitis	4584 (4.7)	5876 (2.2)	13.8		2679 (4.5)	2920 (4.9)	2.3
Conjunctivitis, unspecified	4919 (5.0)	6924 (2.5)	13.0		2828 (4.8)	3153 (5.3)	2.9
Acute suppurative otitis media	5124 (5.2)	359 (0.1)	31.9		483 (0.8)	335 (0.6)	1.6.
Acute nasopharyngitis	23,711 (24.3)	41,787 (15.4)	22.4		12,961 (21.8)	13,468 (22.7)	2.2
Acute sinusitis, unspecified	3465 (3.5)	1618 (0.6)	20.7		1214 (2.0)	1152 (1.9)	0.7
Acute tonsillitis, unspecified	4223 (4.3)	2636 (1.0)	20.9		1711 (2.9)	1671 (2.8)	0.4
Acute laryngopharyngitis	1968 (2.0)	1932 (0.7)	11.3		960 (1.6)	981 (1.7)	0.3
Other acute upper respiratory infections of multiple sites	2095 (2.1)	2574 (0.9)	9.6		1064 (1.8)	1076 (1.8)	0.2
Acute upper respiratory infection, unspecified	18,620 (19.1)	27,484 (10.1)	25.7		9907 (16.7)	10,328 (17.4)	2.0
Pneumonia, unspecified	4583 (4.7)	399 (0.1)	29.8		475 (0.8)	369 (0.6)	1.2
Acute bronchitis	8008 (8.2)	9921 (3.6)	19.4		4082 (6.9)	4307 (7.2)	1.6
Acute bronchiolitis	14,428 (14.8)	4461 (1.6)	49.0		4239 (7.1)	3845 (6.5)	2.5
Allergic rhinitis	1788 (1.8)	2054 (0.8)	9.6		861 (1.4)	912 (1.5)	0.8
Gastro-esophageal reflux disease without esophagitis	2156 (2.2)	4719 (1.7)	3.4		1311 (2.2)	1338 (2.3)	0.3
Functional dyspepsia	1649 (1.7)	4058 (1.5)	1.8		1035 (1.7)	1064 (1.8)	0.4
Noninfective gastroenteritis and colitis, unspecified	3323 (3.4)	6215 (2.3)	6.6		1932 (3.3)	2038 (3.4)	1.1
Constipation	2851 (2.9)	7597 (2.8)	0.8		1785 (3.0)	1878 (3.2)	0.9
Functional intestinal disorder, unspecified	2097 (2.1)	4918 (1.8)	2.6		1280 (2.2)	1318 (2.2)	0.5
Impetigo [any organism] [any site]	1528 (1.6)	2293 (0.8)	6.5		868 (1.5)	923 (1.6)	0.9
Other atopic dermatitis	3406 (3.5)	9143 (3.4)	0.8		2156 (3.6)	2199 (3.7)	0.4
Atopic dermatitis	10,096 (10.3)	26,901 (9.9)	1.7		6317 (10.6)	6500 (10.9)	1.0
Seborrheic infantile dermatitis	1789 (1.8)	5201 (1.9)	0.7		1110 (1.9)	1128 (1.9)	0.2
Seborrheic dermatitis	1357 (1.4)	3815 (1.4)	0.1		837 (1.4)	885 (1.5)	0.7
Diaper dermatitis	6024 (6.2)	15,755 (5.8)	1.7		3722 (6.3)	3749 (6.3)	0.2
Allergic contact dermatitis	2659 (2.7)	6832 (2.5)	1.5		1642 (2.8)	1595 (2.7)	0.5
Irritant contact dermatitis, unspecified cause	1087 (1.1)	2838 (1.0)	0.7		638 (1.1)	642 (1.1)	0.1
Dermatitis	2680 (2.7)	7326 (2.7)	0.3		1661 (2.8)	1653 (2.8)	0.1

Pyogenic granuloma	1693 (1.7)	3211 (1.2)	4.6		1027 (1.7)	1095 (1.8)	1.0
Urinary tract infection, site not specified	2778 (2.8)	693 (0.3)	21.1		603 (1.0)	555 (0.9)	0.7
Cardiac murmur	976 (1.0)	2222 (0.8)	2.1		557 (0.9)	574 (1.0)	0.3
Nausea and vomiting	2595 (2.7)	5606 (2.1)	3.9		1541 (2.6)	1611 (2.7)	0.8
Fever, unspecified	4837 (5.0)	6350 (2.3)	14.1		2150 (3.6)	2093 (3.5)	0.5
Nonspecific symptoms peculiar to infancy	1097 (1.1)	3090 (1.1)	0.0		708 (1.2)	712 (1.2)	0.1
Malnutrition	4 (0.0)	4 (0.0)	0.3		2 (0.0)	2 (0.0)	0.0
Drug (drug classification code) used before 6 months of age							
Sedative- hypnotics	3776 (3.9)	6190 (2.3)	9.1		2128 (3.6)	2285 (3.8)	1.5
Antiepileptics	18 (0.0)	7 (0.0)	1.5		7 (0.0)	5 (0.0)	0.3
Antipyretics	25,600 (26.2)	30,021 (11.0)	39.7		12,247 (20.6)	12,537 (21.1)	1.3
Psycho-nervous system drug	5139 (5.3)	3632 (1.3)	22.1		2046 (3.4)	2126 (3.6)	0.8
Automatic nervous system drugs	31 (0.0)	34 (0.0)	9.2		15 (0.0)	16 (0.0)	0.4
Antispasmodics	1725 (1.8)	2007 (0.7)	9.2		884 (1.5)	911 91.5)	2.9
Antihistamine	43,608 (44.7)	34,826 (12.8)	75.0		20,207 (34.0)	20,928 (35.2)	0.1
Other allergic drug	411 (0.5)	213 (0.1)	7.4		136 (0.2)	133 (0.2)	0.1
Circulatory system drugs	599 (0.6)	277 (0.1)	8.6		167 (0.3)	163 (0.3)	3.1
Respiratory system drugs	54,902 (56.2)	40,861 (15.0)	95.1		25,424 (42.8)	26,224 (44.1)	3.0
Digestive system drugs	54,622 (55.9)	57,403 (21.1)	76.5		26,303 (44.2)	27,111 (45.6)	1.0
Hormone drugs	3869 (4.0)	1193 (0.4)	24.1		929 (1.6)	841 (1.4)	1.0
steroid	3775 (3.9)	954 (0.4)	24.6		871 (1.5)	788 (1.3)	0.3

Abbreviations, N, Number; ICD, International Classification of Diseases; ER, Emergency room. [a] Unless otherwise specified, all baseline characteristics were assessed within the first 3 months of age of participants. [b] Propensity score matching (1:1) was performed to reduce bias for the selection of the comparison group. Matching was performed by the Mahalanobis algorithm with a caliper of 0.01 using multivariable logistic regression with 79 previously chosen covariates as defined in Table S2. [c] Results are reported as N (%) unless otherwise indicated. [d] Differences greater than 10% were interpreted as meaningful differences. All standardized differences of cohort values were < 0.05.

Table S6. Risk of linear and ponderal growth in preschool-age for early antibiotics exposure in the additional cohort[a].

	All Data (*n* = 369,733)				Matched Data (*n* = 118,886)[b]		
	Antibiotic, N (%)		aOR[f] (95% CI)		Antibiotic, *n* (%)		aOR[f] (95% CI)
	Users (*n* = 97,653)	Non-users[c] (*n* = 272,080)			Users (*n* = 59,443)	Non-users[c] (*n* = 59,443)	
**Main outcome - linear growth**							
Stunting [d]	843 (0.86)	2140 (0.79)	**1.144 (1.050 to 1.246)**		500 (0.84)	474 (0.79)	1.052 (0.918 to 1.205)
Short stature [e]	2182 (2.23)	5727 (2.10)	**1.058 (1.030 to 1.086)**		1339 (2.25)	1311 (2.21)	1.073 (0.990 to 1.164)
aβ (95% CI) [g]	**-0.020 (-0.026 to -0.014)**				**-0.024 (-0.033 to -0.015)**		
**Secondary outcome - ponderal growth**							

Obesity [h]	9884 (10.12)	25,153 (9.24)	**1.133 (1.104 to 1.163)**		5901 (9.93)	5639 (9.49)	**1.063 (1.021 to 1.107)**
Overweight i	26,002 (26.63)	68,244 (25.08)	**1.102 (1.084 to 1.120)**		15,513 (26.10)	15,338 (25.80)	**1.029 (1.003 to 1.056)**
aβ (95% CI) [g]	**0.052 (0.044 to 0.059)**				**0.016 (0.005 to 0.028)**		

Abbreviations, N, Number; aOR, adjusted odds ratio; CI, confidence interval; aβ, adjusted β. [a] The main cohort consisted of the antibiotic user group who used antibiotics within the first 6 months of life and non-user group who did not use antibiotics within the first 6 months of life. [b] Propensity score matching (1:1) was performed to reduce bias for the selection of the comparison group. Matching was performed by the Mahalanobis algorithm with a caliper of 0.01 using multivariable logistic regression with 79 previously chosen covariates as defined in Table S2. [c] Indicates the reference group [d] Stunting was defined as a height for age z score < – 2.0. [e] Short stature was defined as a height for age z score < – 1.64. [f] Adjusted odds ratios were assessed using a generalized estimating equation with a binomial distribution logit link function and exchangeable working correlation structure, Adjusting for birthweight, sex, breastfeeding status within 4 to 6 months of age, residence at birth and economic status. [g] The adjusted estimates and 95% CIs of height for age z score and BMI for age z score were as-sessed using a mixed model of GENMOD, adjusting for birthweight, sex, breastfeeding status within 4 to 6 months after birth, residence at birth and economic status. [h] Overweight was defined as a BMI for age z-score ≥ 1.03. [i] Obesity was defined as a BMI for age z score ≥ 1.64. Bold values indicate $p < 0.05$.

Table S7. Subgroup analysis grouped by the status of breastfeeding during the first 4 to 6 months of life, or in a binary fashion by birthweight.

**category**		**Unmatched Data**		**Matched Data [a]**	
		**β (95% CI) [f]**			
		**Main cohort [b]**	**Additional cohort [c]**	**Main cohort [b]**	**Additional cohort [c]**
**HFA z score**	Breastfeeding [d]	**−0.041 (−0.048 to −0.034)**	**−0.027 (−0.038 to −0.016)**	**−0.026 (−0.037 to −0.014)**	**−0.019 (−0.036 to −0.003)**
	Not breastfeeding [d]	**−0.041 (−0.048 to −0.034)**	**−0.022 (−0.032 to −0.012)**	**−0.020 (−0.031 to −0.009)**	**-0.023 (−0.038 to −0.007)**
	Lower half [e]	**−0.046 (−0.530to −0.039)**	**−0.016 (−0.026 to −0.006)**	**−0.029 (−0.040 to −0.018)**	−0.015 (−0.031to 0.000)
	Upper half [e]	**−0.039 (−0.046 to −0.032)**	**−0.027 (−0.038 to −0.017)**	**−0.013 (−0.024 to −0.001)**	**−0.024 (−0.040 to −0.008)**

Abbreviations, HFA, height for age. [a] Propensity score matching (1:1) was performed to reduce bias for the selection of the comparison group. Matching was performed by the Mahalanobis algorithm with a caliper of 0.01 using multivariable logistic regression with 79 previously chosen covariates as defined in Table S2. [b] The main cohort consisted of the antibiotic user group who used antibiotics within the first 6 months of life and the non-user group who did not use antibiotics within the first 6 months of life. [c] The additional cohort consisted of the antibiotic user group who used antibiotics within the first 3 months of life and the non-user group who did not use antibiotics within the first 3 months of life. [d] Data about the status of breastfeeding were obtained by the 1st National health screening program of infants and children at 4 to 6 months of age, which were grouped in a binary fashion to breastfeeding or not. [e] Data about birthweight of participants were obtained by the 1st National health screening program of infants and children at 4 to 6 months of age, which were grouped in a binary fashion to lower half vs upper half (median = 3.24 kg). [f] The estimates and 95% CIs of height for age z score were as-sessed using a mixed model of GENMOD. Bold values indicate $p < 0.05$.

Table S8. Post-hoc analysis for effect on linear growth by routes of antibiotic administration.

**category**	**Route [d]**	**All Data**		**Matched Data [a]**	
		**β (95% CI) [e]**			
		**Main cohort [b]**	**Additional cohort [c]**	**Main cohort [b]**	**Additional cohort [c]**
**HFA z score**	Parenteral	**−0.028 (−0.036 to -0.019)**	0.006 (−0.007 to 0.020)	−0.0126 (−0.0356 to 0.0104)	0.0104 (−0.0109 to 0.0317)
	Per oral	**−0.040 (−0.045 to -0.035)**	**−0.028 (−0.036 to −0.020)**	**−0.0178 (−0.0283 to −0.0072)**	**−0.0246 (−0.0363 to −0.0128)**

Abbreviations, HFA, height for age. [a] Propensity score matching (1:1) was performed to reduce bias for the selection of the comparison group. Matching was performed by Mahalanobis algorithm with a caliper of 0.01 using multivariable logistic regression with 79 previously chosen covariates as defined in Table S2. [b] The main cohort consisted of the antibiotic user group who have been exposed the antibiotics within the first 6 months of age and non-user group without antibiotic exposure within the first 6 months of age. [c] The additional cohort consisted of the antibiotic user group who have been exposed the antibiotics within the first 3 months of age and non-user group without antibiotic exposure within the first 3 months of age. [d] The routes of antibiotic administration were divided into parenteral use (intravenous or intramuscular) and per oral by description of drug prescription. [e] The estimates and 95% CIs of height for age z score were as-sessed using a mixed model of GENMOD. Bold values indicate $p < 0.05$.
